# HIV- 1 Protease Inhibits Cap- and Poly(A)-Dependent Translation upon eIF4GI and PABP Cleavage

**DOI:** 10.1371/journal.pone.0007997

**Published:** 2009-11-24

**Authors:** Alfredo Castelló, David Franco, Pablo Moral-López, Juan J. Berlanga, Enrique Álvarez, Eckard Wimmer, Luis Carrasco

**Affiliations:** 1 Centro de Biología Molecular “Severo Ochoa” (CSIC-UAM), Nicolás Cabrera 1, Universidad Autónoma de Madrid, Cantoblanco, Spain; 2 State University of New York at Stony Brook, Long Island, New York, United States of America; University of California San Francisco, United States of America

## Abstract

A number of viral proteases are able to cleave translation initiation factors leading to the inhibition of cellular translation. This is the case of human immunodeficiency virus type 1 protease (HIV-1 PR), which hydrolyzes eIF4GI and poly(A)-binding protein (PABP). Here, the effect of HIV-1 PR on cellular and viral protein synthesis has been examined using cell-free systems. HIV-1 PR strongly hampers translation of pre-existing capped luc mRNAs, particularly when these mRNAs contain a poly(A) tail. In fact, HIV-1 PR efficiently blocks cap- and poly(A)-dependent translation initiation in HeLa extracts. Addition of exogenous PABP to HIV-1 PR treated extracts partially restores the translation of polyadenylated luc mRNAs, suggesting that PABP cleavage is directly involved in the inhibition of poly(A)-dependent translation. In contrast to these data, PABP cleavage induced by HIV-1 PR has little impact on the translation of polyadenylated encephalomyocarditis virus internal ribosome entry site (IRES)-containing mRNAs. In this case, the loss of poly(A)-dependent translation is compensated by the IRES transactivation provided by eIF4G cleavage. Finally, translation of capped and polyadenylated HIV-1 genomic mRNA takes place in HeLa extracts when eIF4GI and PABP have been cleaved by HIV-1 PR. Together these results suggest that proteolytic cleavage of eIF4GI and PABP by HIV-1 PR blocks cap- and poly(A)-dependent initiation of translation, leading to the inhibition of cellular protein synthesis. However, HIV-1 genomic mRNA can be translated under these conditions, giving rise to the production of Gag polyprotein.

## Introduction

Viruses rely on cellular machinery to synthesize their proteins since this complex process requires numerous components that cannot all be encoded by viral genomes. Thus, viral mRNAs have to compete with host mRNAs for ribosomes and other components of the translation machinery [1]. To achieve this goal, viruses have evolved sophisticated mechanisms to maximize the translation of their mRNAs. Since the initiation of translation is important in the regulation of gene expression in eukaryotic cells, cytolytic viruses usually target this step to ensure the synthesis of viral proteins [2]. A number of viral proteases are involved in the proteolysis of translation initiation factors, such as eIF4G and PABP [2], [3]. Under these conditions the association of host mRNAs with ribosomes is severely impaired, whereas viral mRNAs can efficiently interact with the translation machinery [1], [2].

eIF4G mediates the formation of the translation initiation complex by acting as a scaffold protein that physically links the 40S ribosomal subunit with the mRNA [2], [4]. In the canonical initiation process of translation, the cap structure and the poly(A) tail of mRNAs are recognized and joined by eIF4E and PABP, respectively [5], [6]. In turn, both proteins interact with the N-terminal portion of eIF4G, which recruits the small ribosomal subunit to the proximity of the mRNA by the interaction of its C-terminal domain with eIF3 [2], [7]. In addition, eIF4G contains binding sites for other proteins implicated in translation such as eIF4A and the protein kinase Mnk1 [2]. A number of viruses such as certain picornaviruses, retroviruses and caliciviruses, encode proteases which hydrolyze eIF4G, and separate the domain implicated in mRNA recognition (N-terminal domain) from the portion involved in the recruitment of 40S ribosomal subunit (C-terminal domain) [1], [2], [3], [8], [9]. For example, the association of host mRNAs and ribosomes is impaired in poliovirus (PV) infected cells by eIF4G cleavage, while viral mRNA can interact with the translation machinery by means an internal ribosome entry site (IRES) placed in its 5′ untranslated region (5′ UTR) [10], [11]. We previously described that eIF4GI is cleaved in HIV-1-infected cells, with HIV-1 PR being responsible for this event [9]. In fact, IRES elements have been identified within HIV-1, HIV-2, simian immunodeficiency virus and feline immunodeficiency virus genomic mRNAs [12], [13], [14], [15]. However, little is known about the regulation of retroviral IRES-driven translation by cellular and viral factors.

Cleavage of PABP by viral proteases has been described recently [16], [17], [18], [19], [20], [21]. PABP binds to the poly(A) tail present at the 3′ end of mRNAs [7]. This protein directly participates in the initiation of translation by linking the poly(A) tail of mRNAs to eIF4G [5]. The N-terminal domain of PABP (NTD) contains four RNA recognition motifs (RRM) and the eIF4G-binding site, while the C-terminal domain (CTD) interacts with eIF4B and eukaryotic release factor 3 (eRF3) [22], [23], [24], [25] and regulatory proteins such as PABP-interacting protein 1 and 2 (Paip 1 and 2) [26], [27], [28], and mediates the oligomerization of PABP on the poly(A) tail [7]. PV 3C^pro^ and, to a lesser extent, 2A^pro^, cleave PABP separating NTD and CTD [18]. Proteolysis of PABP by 3C^pro^ impairs poly(A)-dependent initiation of translation [19]. Proteases from both human immunodeficiency virus (HIV)-1 and 2 also cleave PABP at two distant positions; one located at the NTD and CTD junction and another within RRM3 [16]. A previous work has investigated the effect of PABP cleavage by PV proteases on protein synthesis [19], but the action of HIV-1 PR on poly(A)-dependent translation remains unexplored.

The cap structure and poly(A) tail synergistically enhance translation [2], [7]. In this regard, eIF4E and PABP interaction with eIF4G induces a circular mRNA conformation, which might enhance ribosome recycling [29]. On the other hand, the interaction between eIF4G and PABP could induce conformational changes in the initiation complex in turn increasing the affinity of eIF4E for the cap structure [30]. Thus, the hydrolysis of eIF4G or PABP could inhibit the synergism provided by the cap and poly(A) tail. In this work we analyze the contribution of the cleavage of eIF4GI and PABP by HIV-1 PR to the inhibition of translation directed by cap and poly(A) tail of mRNAs in cell-free systems and their impact on picornavirus IRES-driven translation. In addition, the effects of HIV-1 PR on the translation of HIV-1 genomic mRNA have been also examined.

## Materials and Methods

### Plasmid Construction and In Vitro Synthesis of mRNAs

pKS-luc, pTM1-luc and pT5′NCP-luc were used as a template to synthesize luciferase (luc) mRNAs, EMCV and PV IRES-containing luc mRNA, respectively. These plasmids were described in previous reports [31], [32], [33], [34]. Human globin 5′UTR-containing luc mRNAs were obtained by *in vitro* transcription using pKS-GL-FL as a template. This plasmid was kindly provided by Drs. M. Hentze and F. Moretti (EMBL, Heidelberg, Germany). The *in vitro* transcription was carried out with T7 polymerase (Promega) according to the indications of the manufacturer and using GpppG or GpppA (New England Biolabs). *In vitro* polyadenylation was performed with poly(A) polymerase (New England Biolabs) and tested by agarose gel electrophoresis. The mRNA was purified using the *Chroma spin columns kit* (BD Biosciences). The amount of mRNA was analyzed with the NanoDrop ND-1000 spectrophotometer. The plasmid pGEX-2T-PABP1 containing the sequence encoding the human PABP1, lacking the first nine amino acids and fused to the GST (glutathione S-transferase) gene, was obtained as described previously [35] and was kindly provided by A. Nieto (Centro Nacional de Biotecnología, CSIC, Madrid, Spain). pKS-HIV-1 was obtained by digestion of pBH10 and pKS plasmid with *Sac I*, followed by a treatment with T4 ligase.

### In Vitro Translation

The HeLa S3 extracts and the translation reaction mix were obtained as previously described [36], [37], [38]. Krebs-2 extracts and the respective reaction mix were obtained as previously described [39], [40], [41]. Nuclease-treated RRL (Promega) was used according to the manufacturer's instructions. 20 ng (1.3 ng/µl) of HIV-1 PR or 1 µg (66 ng/µl) of maltose binding protein (MBP)-2A^pro^ were added to translation mix as indicated in the figure legends. Protein synthesis was analyzed by metabolic labelling with 50 µCi of [^35^S]Met-[^35^S]Cys/ml (Promix; Amersham Biosciences), followed by SDS-PAGE, fluorography and autoradiography. The samples used to measure luciferase (Luc) activity were recovered in luciferase lysis buffer (see below), whereas sample buffer was added to the replicates to be analyzed by SDS-PAGE followed by Western blotting.

### Real-Time RT-PCR

The levels of luc mRNAs in cellular lysates were determined by real-time quantitative RT-PCR. Total RNA was extracted from *in vitro* translation at the times indicated in each figure using the RNeasy commercial kit (Qiagen), according to the manufacturer's recommendations. The primers luc-forward (5′ -GAACGAGGACGGAGATGTCATCG-3′) and luc-reverse (5′- GCTCCTCTTCTGGTATTCTTGGCG - 3′) were used to quantify luc RNAs with Master SYBR Green I Kit (Roche Diagnostics), following the protocol previously described [42]. To validate the results using these primers, real-time RT-PCR using a Taqman probe designed by Applied Biosystems was carried out as previously indicated [43]. As a control, 18 S rRNA was measured using the Hs 99999901-m1 assay (Applied Biosystems). The amount of the different luc mRNAs was determined by taking into consideration the 18 S rRNA levels [43]. Data analysis was carried out with the SDS-7000 software (Version 1.1).

### Measurement of Luciferase Activity

Extracts were recovered in a buffer containing 25 mM glycylglycine (pH 7.8), 0.5% Triton X-100 and 1 mM dithiothreitol. Luc activity was determined using *luciferase assay system* (Promega) and Mononlight 2010 apparatus (Analytical Luminescence Laboratory) as described previously [8], [9].

### Immunoblotting

Western blot analysis was carried out using a rabbit antibodies mix against the N-terminal and C-terminal portion of the initiation factors eIF4GI [44] and eIF4GII (a generous gift from N. Sonenberg, McHill University, Montreal, Canada) at 1∶1000 dilution. PABP was detected using a monoclonal antibody (Abcam) at 1∶300 dilution or a rabbit polyclonal antibody raised against GST-PABP at 1∶3000 dilution.

### Purification of Recombinant Proteins

HIV-1 PR was provided by I. Pichova (Centralized Facility for AIDS Reagents). The chimeric MBP (maltose-binding protein) and MBP–2A^pro^ were purified by affinity chromatography, as described previously [45]. The pGEX-2T and pGEX- 2T-PABP1 plasmid was used to purify the GST and GST–PABP1 protein, respectively, by affinity chromatography, using a glutathione–agarose 4B resin (Amersham Biosciences) as described previously [35].

### Statistical Analysis

Luc activity data and luc RNA levels are presented as mean values±SD. Differences were tested for significance by means of the Student *t*-test. In each experiment, protease treated extracts were compared with respect to the control. A probability level *P*<0.05 was considered significant.

## Results

### Effect of HIV-1 PR on the Translation of Pre-Existing Exogenous mRNAs in HeLa Extracts

**Figure 1:**
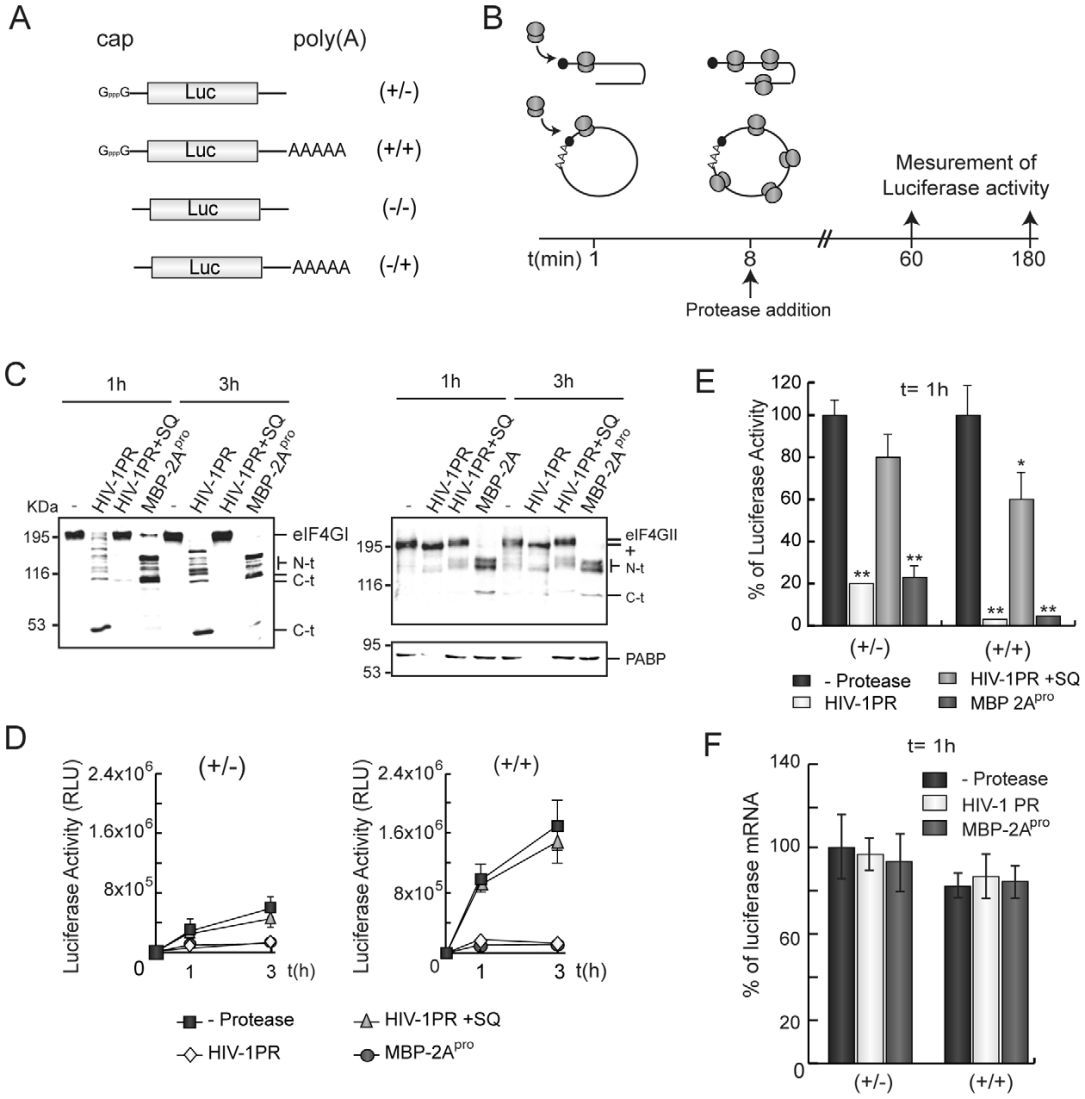
Translation reinitiation of luc mRNAs in HIV-1 PR treated HeLa extracts. A) Schematic representation of (+/−), (+/+), (−/+) and (−/−) luc mRNAs. B) HeLa extracts were programmed with 50 ng of (+/−) or (+/+) luc mRNAs. To ensure that exogenous mRNAs are engaged in protein synthesis machinery, viral proteases (20 ng of HIV-1 PR or 1 µg of MBP-2A^pro^) were added to the lysates 8 min later. Luciferase activity was then analyzed 1 and 3 h after the initiation of the reaction. C) eIF4GI, eIF4GII and PABP were detected by western blot. D) Luc activity was measured at each time point. Error bars indicate standard deviations (SD) obtained from three measurements of each sample. E) Representation of the percentage of Luc activity obtained from HeLa extracts programmed with (+/−) or (+/+) mRNAs in presence of HIV-1 PR or MBP-2A^pro^ with respect to control extracts after 1 h of incubation. SDs were obtained from three independent experiments. F) In parallel, RNAs were isolated after 1 h and quantified by real-time RT-PCR. Relative luc RNA levels are represented. KDa, molecular weights markers. N-t, N-terminal proteolysis fragments of eIF4GI or eIF4GII; C-t, C-terminal fragments of eIF4GI or eIF4GII; RLU, relative light units. +, HIV-1 PR-modified eIF4GII. *P<0.05; **P<0.01.

The cleavage of eIF4GI and PABP by HIV-1 PR has been recently described [9], [16]. Since a circular mRNA conformation, induced by the interaction between eIF4G and PABP, might enhance ribosome recycling after the termination of translation, we analyzed the effects of HIV-1 PR on pre-existent capped/non-polyadenylated (+/−) or capped/polyadenylated (+/+) luc mRNAs (Figure 1A). As a control, the recombinant protein MBP tagged to PV 2A^pro^ (MBP-2A) was also used [45], [46]. HeLa extracts were chosen to carry out this experiment since they support a strong cap-poly(A) synergism [19]. Tanking into account the translatability of (+/+), (+/−), uncapped (−/−) and uncapped polyadenylated (−/+) luc mRNAs (Figure 1A and Figure S1A), we calculated that the cap-poly(A) synergism supported by our extracts was of about 5-fold.

Many host mRNAs are engaged in protein synthesis machinery prior to virus infection. Therefore, these mRNAs must be stripped from ribosomes to ensure high levels of viral protein synthesis. To induce similar conditions in HeLa extracts, (+/−) and (+/+) luc mRNAs were added 8 min before viral proteases (estimated time for the synthesis of a Luc molecule [19]) and luciferase activity was measured after 1 and 3 h (See scheme in Figure 1B). MBP-2A^pro^ efficiently cleaved eIF4GI and eIF4GII 1 h after addition, while PABP remained largely intact (Figure 1C). In contrast, HIV-1 PR proteolyzed both eIF4GI and PABP at this time. As previously observed [16], cleavage products from PABP proteolysis were not detected in these extracts with the monoclonal antibody (Abcam) (Figure 1C). This result suggests that these cleavage products are unstable in lysates [16], [19]. In extracts incubated with HIV-1 PR, the electrophoretic mobility of eIF4GII was slightly increased, probably due to cleavage at the N-terminal end of the protein (Figure 1C). In fact, double treatment of HeLa extracts with 2A^pro^ and HIV-1 PR renders an eIF4GII C-terminal polypeptide similar to that found with 2A^pro^ alone, whereas the N-terminal fragment increased its electrophoretic mobility (data not shown). This proteolysis might not be very relevant for translation since the functional domains in the N-terminus of eIF4GII should remain intact [47].

After 10 min of (+/+) luc mRNA addition, a significant value of luciferase activity was detected (Figure S1C), which is coherent with the time predicted for the synthesis of one Luc molecule [19]. Moreover, this result suggests that luc mRNAs were engaged in protein synthesis machinery before protease addition. Translation of (+/−) and (+/+) luc mRNAs was severely impaired in the extracts treated with MBP-2A^pro^ or HIV-1 PR, although the substrates cleaved by each protease differ (Figure 1D). Consistent with this, MBP protein did not inhibit translation of luc mRNAs (data not shown). Addition of 2.5 µM saquinavir (SQ), a potent inhibitor of HIV-1 PR, blocked the action of retroviral protease on initiation factors, as well as on translation (Figure 1C and D). Notably, the inhibition of Luc synthesis from (+/+) mRNA was higher than from its (+/−) counterpart (Figure 1E). In fact, the amount of Luc activity detected from protease treated extracts programmed with either (+/−) or (+/+) mRNAs was similar, irrespective of the protease employed (Figure 1D). These data indicate that HIV-1 PR, as occurs with MBP-2A^pro^, can inhibit cap- and poly(A)-dependent translation, reducing Luc activity to a basal level. To test the state of luc mRNAs in HeLa extracts, these mRNAs were quantified by real-time RT-PCR with specific primers designed against luc sequence. The amount of both (+/−) and (+/+) luc mRNAs was similar in each case after 1 h of incubation, irrespective of protease addition (Figure 1F). The results obtained using a Taqman probe designed against other regions of luc gene sequence were similar (data not shown). Thus, mRNA stability was not responsible for those effects. In addition, translation of pre-existing exogenous as well as endogenous mRNAs was strongly inhibited by HIV-1 PR in other cap-poly(A) synergistic extracts such as non nuclease-treated Krebs-2 lysates (Figure S2). In particular, translation of (+/+) luc mRNA was more susceptible to protease treatment than that observed for (+/−) luc mRNA (Figure S2A and B). Rabbit reticulocyte lysates (RRL) treated with nuclease do not exhibit cap-poly(A) synergism [19], [48]. In contrast to the results observed in cap-poly(A) synergistic extracts, MBP-2A^pro^ and HIV-1 PR blocked translation of (+/+) and (+/−) luc mRNAs in RRL to a similar extent (data not shown). Taking together the results obtained in the three different cell-free systems (HeLa, Krebs-2 and RRL), we can hypothesize that HIV-1 PR inhibits efficiently ongoing translation of luc mRNAs by means of the specific inhibition of cap- and poly(A)-dependent translation.

### HIV-1 PR Blocks Cap- and Poly(A)-Dependent Translation Initiation

**Figure 2:**
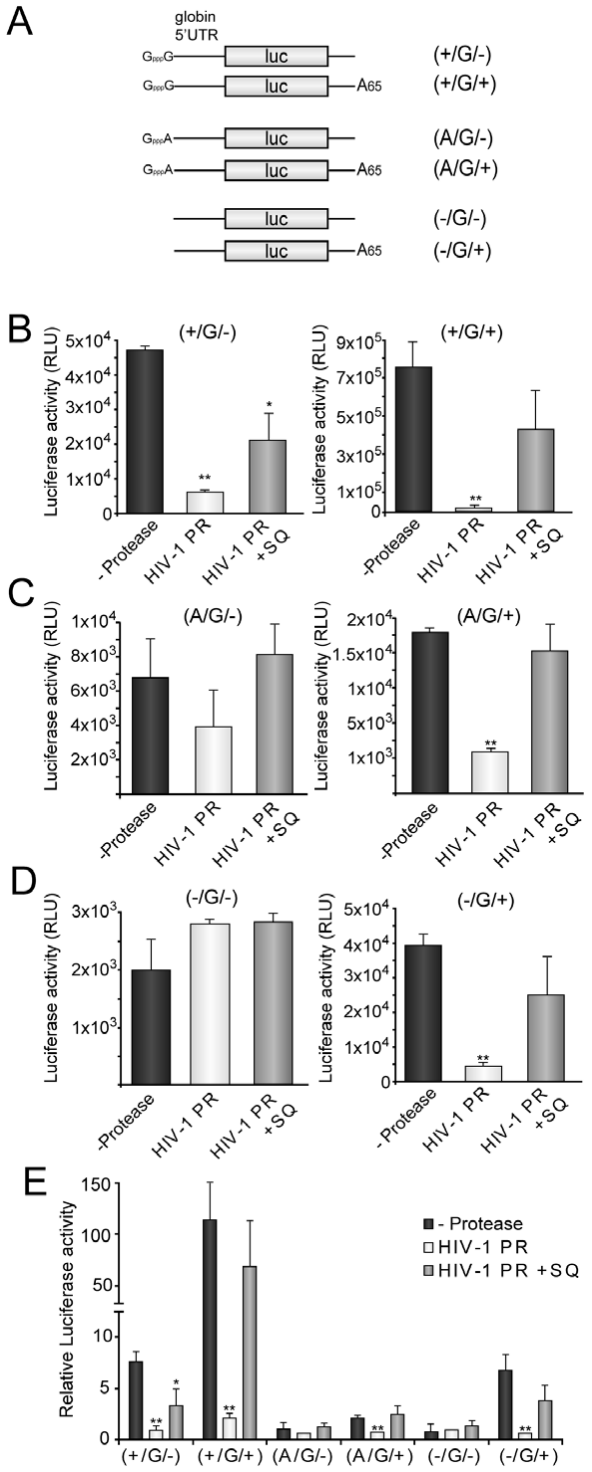
Analysis of cap- and poly(A)-dependent translation of luc mRNAs in HIV-1 PR treated HeLa extracts. HeLa extracts were treated with 20 ng HIV-1 PR. After 30 min, SQ 2.5 µM was added to the lysate. Simultaneously, extracts were programmed with 20 ng (+/G−), (+/G/+), (A/G/−), (A/G/+), (−/G/−) or (−/G/+) luc mRNAs, which are schematized in panel (A). As a control, a replicate was incubated with 2.5 µM SQ from the beginning of the reaction (grey bars). B, C and D) Luc activity was analyzed 30 min later and the data obtained from translation of each mRNA were plotted. E) Relative Luc activity obtained from each mRNA in presence and absence of HIV-1 PR was represented. S.D. were obtained from three independent experiments. *P<0.05; **P<0.01.

Our next goal was aimed to determine whether PABP and eIF4GI cleavage hamper the participation of cap and poly(A) tail in the initiation of translation. To this end, we used a new set of luc mRNAs that resemble the behaviour of cellular mRNAs since they contain the 5′ UTR of human globin mRNA placed before luc ORF. These mRNAs were capped with normal cap structure (GpppG), with a cap analog that is not able to contribute in initiation of translation (GpppA) or were not capped. In addition, the *in vitro* transcription of the template plasmid lead to the production of a reporter with a poly(A) tail of 65A or lacking this structure [ See Scheme of (+/G/−), (+/G/+), (A/G/−), (A/G/+), (−/G/−) and (−/G/+) in Figure 2A].

HeLa extracts were pre-treated for 30 min with 20 ng of HIV-1 PR and replicates were recovered at this point to analyze the integrity of initiation factors. As expected, eIF4GI and PABP were substantially proteolyzed by HIV-1 PR at this time point (data not shown). SQ was then added to inhibit HIV-1 PR activity while control extracts were incubated for the entire time course with this compound. Next, extracts were programmed with 20 ng of each mRNAs and, finally, luciferase activity was analyzed 1 h later. Cap (GpppG) and poly(A) structures [(+/G/−), (A/G/+) and (−/G/+)] enhanced translation of these reporters by about 7- and 6- to 2-fold respectively (Figure 2B, C, D and E), whereas GpppA (A/G/−) did not contribute to initiation of translation (Figure 2C and E). In addition, simultaneous presence of cap and poly(A) (+/G/+) increase translatability of luc mRNAs by about 114-fold (Figure 2B and E), leading to a translational synergism between both structures of about 12-fold (Figure S1B). HIV-1 PR strongly inhibited the translation of (+/G/−) luc mRNA leading to a similar value of luciferase activity to that obtained from (A/G/−) and (−/G/−) luc mRNAs (∼6-fold inhibition) (Figure 2B and E). In fact, translation of both (A/G/−) and (−/G/−) luc mRNAs was not significantly affected by HIV-1 PR (Figure 2C, D and E). Taking together, these results indicate that HIV-1 PR specifically blocks cap-dependent initiation of translation. Similarly, translation of (A/G/+) and (−/G/+) luc mRNAs was significantly inhibited by HIV-1 PR (∼5-fold inhibition), exhibiting the same translatability as their unpolyadenylated counterparts [(A/G/−) and (−/G/−)] under such conditions (Figure 2C, D and E). Thus, HIV-1 PR also blocks specifically poly(A)-dependent initiation of translation. Finally, (+/G/+) luc mRNA exhibited the highest translatability due to the cap-poly(A) tail synergism supported by HeLa extracts (Figure 2B and Figure S1B). HIV-1 PR deeply inhibited the translation of this mRNA, suggesting that this protease is able to disrupt the cap-poly(A) synergism (Figure 2B).

**Figure 3:**
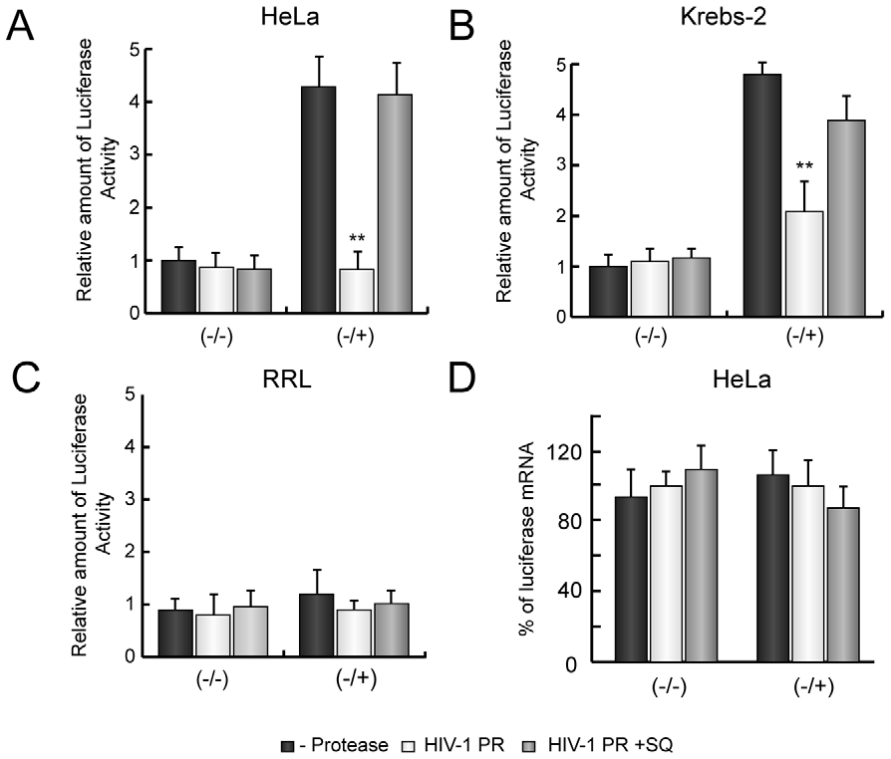
Analysis of poly(A)-dependent translation of luc mRNAs in HIV-1 PR treated HeLa extracts. HeLa (A), Krebs-2 (B) extracts or RRL (C) were treated with 20 ng HIV-1 PR for 30 min. Next, SQ 2.5 µM was added to the translation reaction to inhibit the protease activity. At this time point, extracts were programmed with 200 ng (−/−) or (−/+) mRNAs. As a control, a replicate reaction was incubated with 2.5 µM SQ from the beginning of incubation (grey bars). Luc activity was analyzed 30 min later and relative Luc activity from two independent experiments was represented. D) In parallel, RNAs were isolated from HeLa extracts and the amount of luc mRNA was determined by real-time RT-PCR. Relative luc mRNA levels were then represented. *P<0.05; **P<0.01.

To further reinforce the idea that poly(A)-dependent translation is specifically blocked by HIV-1 PR, we compared the translatability of (−/−) and (−/+) luc mRNAs (see scheme in Figure 1A) in HeLa and Kreb-2 extracts with RRL, because nuclease-treated RRL do not exhibit poly(A)-dependent stimulation of translation [19], [48]. Translation of luc mRNAs was enhanced 4- to 5-fold by the presence of the poly(A) tail in HeLa and Kreb-2 extracts (Figure 3A and B). By contrast, poly(A) tail did not contribute substantially to initiation of translation in RRL (Figure 3C). HIV-1 PR strongly blocked translation of (−/+) luc mRNA without affecting (−/−) translatability in HeLa and Krebs-2 extracts (Figure 3A and B). However, HIV-1 PR was not able to hamper translation of (−/+) luc mRNA in RRL (Figure 3C). To rule out the possibility that mRNA stability could be the cause of the differences between both types of cell free system, luc mRNA levels were determined by real-time RT-PCR after 1 h of incubation in HeLa extracts. Amounts of luc mRNAs were similar irrespective of the presence of poly(A)-tail or HIV-1 PR addition (Figure 3D), suggesting that their stability was the same under the different conditions. These data support the concept that HIV-1 PR specifically blocks poly(A)-dependent initiation of translation.

### Restoration of Poly(A)-Dependent Translation Initiation by Addition of Exogenous PABP

**Figure 4:**
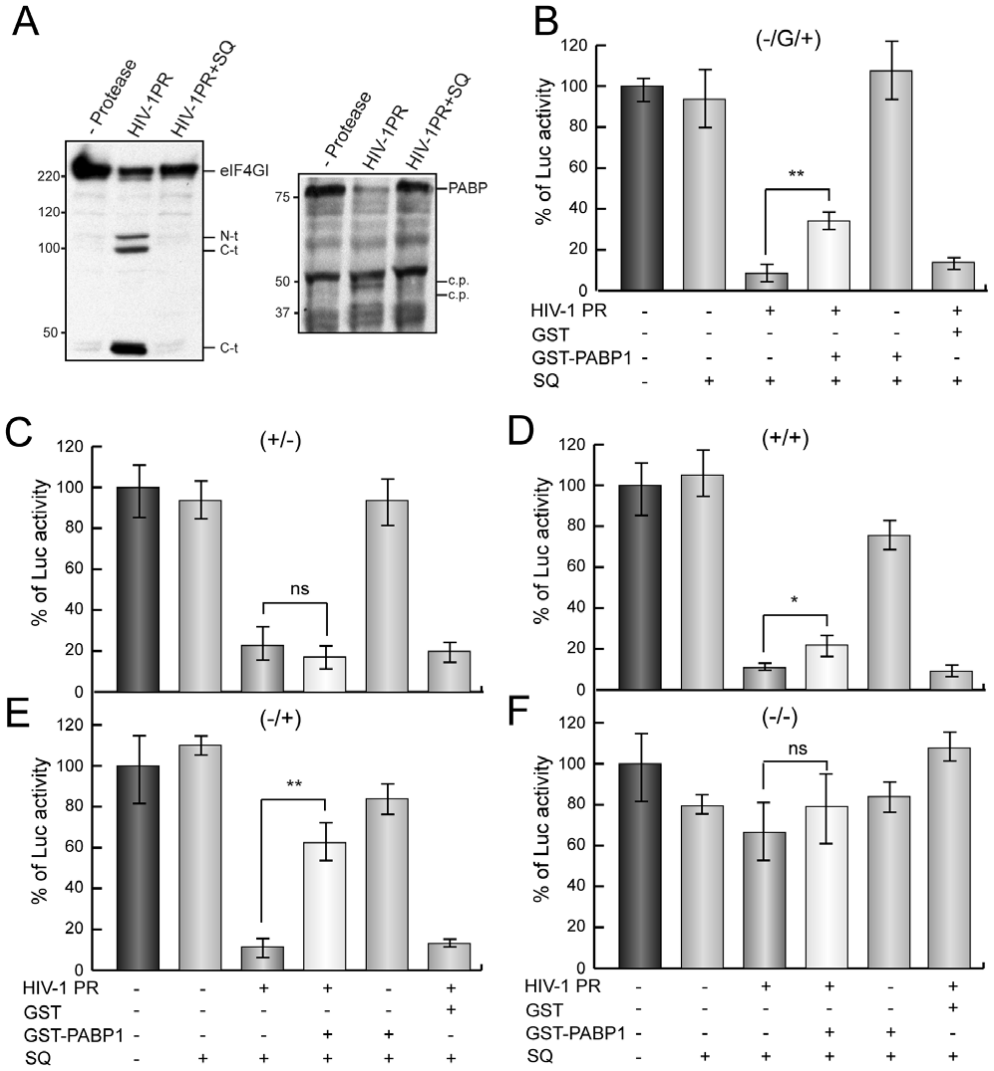
Restoration of poly(A)-dependent translation by addition of recombinant GST-PABP1 in HeLa extracts treated with HIV-1 PR. HeLa lysates were incubated with or without 10 ng HIV-1 PR. The integrity of eIF4GI and PABP was determined by western blotting (A). After 30 min, 20 ng (−/G/+) (B), (+/−) (C), (+/+) (D), (−/+) (E) or (−/−) (F) luc mRNAs and 2.5 µM SQ was added to the extract. In a control reaction, SQ was present throughout the time course (bar 2). The extracts indicated were then supplemented with 25 ng GST-PABP1 or GST. Luc activity was measured 1 h later. The relative Luc activity obtained from (−/G/+) (B), (+/−) (C), (+/+) (D), (−/+) (E) or (−/−) (F) luc mRNAs was represented. *P<0.05; **P<0.01; ns, non-significant; C.p., putative cleavage product.

To determine whether the cleavage of PABP is sufficient to inhibit poly(A)-dependent translation in HIV-1 PR-treated extracts, we examined the effects of addition of exogenous PABP. HeLa extracts were incubated with 10 ng HIV-1 PR for 30 min, because this treatment induces the total cleavage of PABP while eIF4GI is only partially proteolyzed (Figure 4A). The polyclonal antibody against PABP detected weak PABP-derived cleavage products of about 50 and 40 KDa, which are coherent with previous findings [16]. SQ was then added to block protease activity and extracts were programmed with 20 ng of (−/G/+), (+/−), (+/+), (−/+) or (−/−)luc mRNAs (Figure 1A and 2A). Simultaneously, extracts were supplemented or not with 25 ng of GST-PABP1 or GST alone as a control and luciferase activity was measured 1 h later. Luciferase activity decreased strongly (80–90%) in extracts treated with HIV-1 PR programmed with either type of mRNAs (Figure 4B, C, D and E, third bar), but not in the case of (−/−) luc mRNA. These data indicate that cap- as well as poly(A)-dependent translation was inhibited by the retroviral protease as observed in Figure 2. GST addition did not substantially affect the translatability of luc mRNAs in HIV-1-treated extracts (Figure 4B, C, D and E, sixth bar). Addition of GST-PABP1 did not prevent the effect of HIV-1 PR on (+/−) luc mRNA translation, pointing to the idea that PABP does not counteract the inhibition of translation of capped mRNAs without poly(A) tail induced by the retroviral protease (Figure 4C, fourth bar). Notably, 25 ng GST-PABP1 moderately but significantly restored Luc synthesis from (−/G/+) and (−/+) luc mRNA in HIV-1 PR treated extracts (Figure 4B and E, fourth bar). These results further support the idea that PABP cleavage inhibits poly(A)-dependent translation. Finally, GST-PABP addition slightly but significantly recovered translation of (+/+) luc mRNA (Figure 4D, fourth bar). This result is coherent with the idea that eIF4GI cleavage also blocks cap-dependent translation, most probably disrupting the synergism between cap and poly(A) tail. SQ prevented all the effects induced by HIV-1 PR when added at the beginning of this reaction (Figure 4B, C, D and E second bar). Therefore, exogenous PABP specifically re-establishes poly(A)-dependent translation to some extent in lysates incubated with HIV-1 PR. Therefore, our data indicate that exogenous PABP is able to partially rescue the translation driven by poly(A) tail when endogenous PABP is cleaved by HIV-1 PR.

### Effects of HIV-1 PR on Translation of mRNAs Containing IRES and Poly(A) Tail

**Figure 5:**
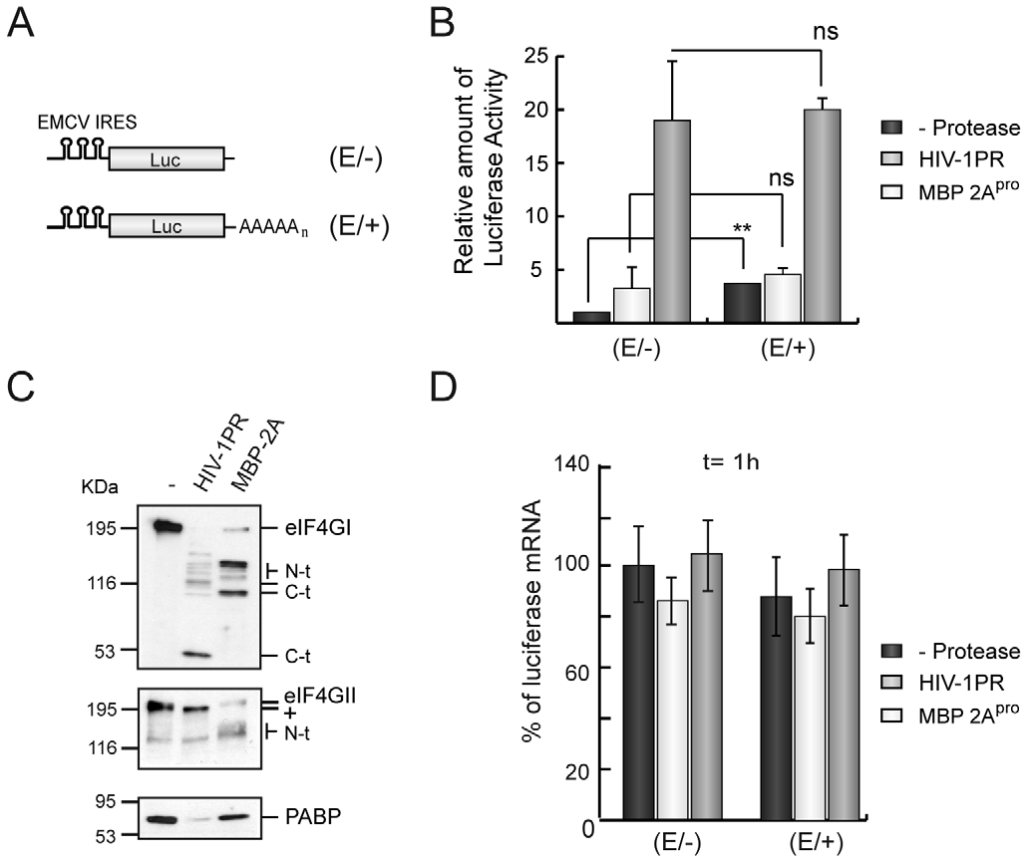
Effects of HIV-1 PR on translation of polyadenylated EMCV-IRES containing mRNAs in HeLa extracts. HeLa extracts were treated with 20 ng of HIV-1 PR or 1 µg of MBP-2A^pro^ for 30 min. HeLa extracts were then programmed with 50 ng of (E/−) or (E/+) luc mRNAs. The samples were analyzed 1 h after the addition of luc mRNAs. A) Schematic representation of (E/−) and (E/+) luc mRNAs. B) Relative amounts of Luc activity. SDs were determined from three measurements of two independent experiments. *P<0.05; **P<0.01; ns, non-significant. C) eIF4GI, eIF4GII and PABP were analyzed by western blot. D) In parallel, after 1 h of translation reaction, total RNA was isolated from each sample and quantified by real-time RT-PCR. The relative levels of luciferase mRNAs were plotted.

Translation of picornavirus mRNAs is stimulated by poly(A) tail to a similar extent to that observed with capped host mRNAs [43], [49], [50]. Nevertheless, hydrolysis of eIF4G by PV 2A^pro^ stimulates encephalomyocarditis virus (EMCV) IRES-driven translation despite the impairment of poly(A)-dependent translation [43], [49], [50]. Although the contribution of eIF4G cleavage by PV 2A^pro^ to translation of picornavirus mRNAs has been extensively studied, the repercussion of PABP hydrolysis on protein synthesis directed by IRES remains unexplored. To this end, HeLa extracts were pre-treated with HIV-1 PR or MBP-2A^pro^ as a control. After 30 min, extracts were programmed with unpolyadenylated EMCV IRES-containing luc mRNA (E/−) or its polyadenylated counterpart (E/+) (Figure 5A). The poly(A) tail increased Luc expression from EMCV-containing mRNAs by about 4–5 fold in HeLa extracts (Figure 5B), whereas almost no stimulation appeared in RRL (data not shown). Translation of (E/−) luc mRNAs increased after HIV-1 PR treatment as compared to control extracts (Figure 5B), indicating that PABP cleavage does not abolish EMCV IRES-driven translation, at least when eIF4GI is also proteolyzed (Figure 5C). Notably, translatability of (E/+) luc mRNA was similar in presence or absence of HIV-1 PR. This result suggests that enhancement of EMCV IRES-driven translation by HIV-1 PR is sufficient to replace poly(A)-dependent translation. A similar result was observed using another mRNA bearing PV IRES (data not shown). Thus, stimulation of IRES-driven Luc synthesis promoted by eIF4GI cleavage counteracts the inhibition of poly(A)-dependent translation induced by PABP proteolysis. Addition of MBP-2A^pro^ to HeLa extracts provoked substantial hydrolysis of both forms of eIF4G, while PABP remained intact (Figure 5C). In MBP-2A^pro^-treated extracts, translation of (E/−) or (E/+) luc mRNAs was strongly stimulated (Figure 5B, grey bars). Nevertheless, the translational enhancement induced by PV 2A^pro^ was lower with (E/+) than with (E/−) luc mRNAs (5-fold versus 18-fold, respectively). Luc activity from MBP-2A^pro^ treated extracts was similar in both cases regardless of whether a poly(A) tail was present (Figure 5B, grey bars). Translation stimulation conferred by poly(A) tail is probably abrogated in MBP-2A^pro^ treated lysates by cleavage of eIF4GI and eIF4GII and compensated by the increase of IRES-driven translation. To determine the amount of IRES-containing luc mRNAs in these extracts, real-time RT-PCR with specific primers against luc sequence was carried out. Of interest, levels of luc mRNA were similar in each case after incubating for 1 h in HeLa extracts (Figure 5D). These results reflect that the differential enhancement on IRES-driven translation observed in extracts treated with HIV-1 PR or MBP-2A^pro^ was not due to changes in the stability of luc mRNAs.

### Impact of HIV-1 PR on the Translation of HIV-1 Genomic mRNA

**Figure 6:**
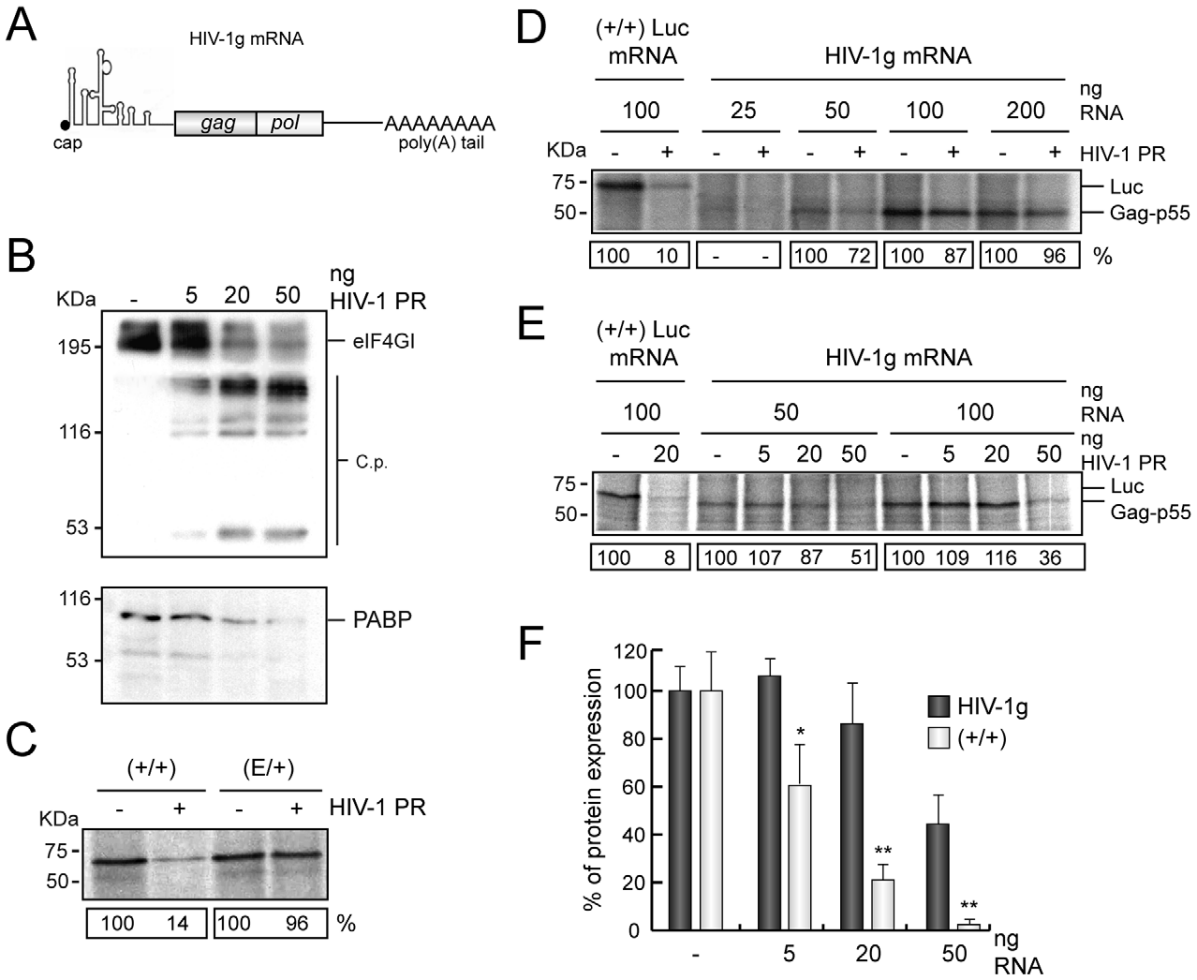
Translation of HIV-1g mRNA in presence of HIV-1 PR. A) Schematic representation of HIV-1g mRNA, indicating the structures of the leader sequence and the open reading frames encoded. B) HeLa extracts were programmed with different doses of HIV-1 PR (5, 20 and 50 ng). eIF4GI and PABP were analyzed by western blot after 30 min of incubation. C) HeLa extracts supplemented with [^35^S]Met-[^35^S]Cys were pre-incubated with 20 ng HIV-1 PR for 30 min. Extracts were then incubated with 2.5 µM SQ and programmed with 100 ng (+/+) or (E/+) luc mRNAs. Translation reaction was stopped after 1 h and protein synthesis was analyzed by SDS-PAGE followed by fluorography and autoradiography. D) HeLa extracts supplemented with [^35^S]Met-[^35^S]Cys were pre-incubated with 20 ng HIV-1 PR for 30 min. Extracts were then incubated with 2.5 µM SQ and subsequently programmed with 20, 50 100 or 200 ng HIV-1g mRNA or 100 ng (+/+) luc mRNA. Translation reaction was stopped after 1 h and protein synthesis was analyzed by SDS-PAGE followed by fluorography and autoradiography. The data shown in this figure is a representative experiment of a set of three independent experiments. E) HeLa extracts supplemented with [^35^S]Met-Cys were pre-incubated with 5, 20 or 50 ng HIV-1 PR for 30 min. 2.5 µM SQ was added to the reaction mixture and next, extracts were programmed with 50 or 100 ng HIV-1g mRNA or 100 ng (+/+) luc mRNA. Translation reaction was stopped after 1 h and protein synthesis was analyzed by SDS-PAGE followed by fluorography and autoradiography. The data shown is a representative experiment of a set of two independent experiments. F) Comparative representation of Gag or Luc synthesis after pre-incubation with increasing amounts of HIV-1 PR (5, 20 and 50 ng). SD, were obtained from two independent experiments. *P<0.05; **P<0.01.

HIV-1 genomic mRNA bears an IRES that comprises its 5′ UTR and part of the coding sequence [12], [13]. However, the exact mechanism by which this mRNA is translated in infected cells is poorly understood. There are at least three viral factors that could influence the translatability of HIV-1 mRNAs: HIV-1 PR, Gag polyprotein and Rev [9], [47], [51], [52], [53]. To determine whether translation of HIV-1 genomic (HIV-1g) mRNA (scheme in Figure 6A) takes place when PABP and eIF4GI have been cleaved by HIV-1 PR, HeLa extracts were treated with this protease for 30 min. SQ was then added to the reaction mixture to inhibit the retroviral protease. Pre-treated extracts were programmed with different amounts of HIV-1g mRNA transcribed, capped and polyadenylated *in vitro*. As a control, translation of (+/+) and (E/+) luc mRNAs was also assayed. After incubating for 1 h, labelled proteins were analyzed by SDS-PAGE, followed by autoradiography. Treatment with 20 ng HIV-1 PR leads to cleavage of eIF4GI and PABP (Figure 6B). As expected, Luc synthesis from (+/+) mRNA was potently inhibited by HIV-1 PR pre-treatment, whereas translation of (E/+) luc mRNA was not affected under these conditions (Figure 6C) as observed above in Figure 5B. Synthesis of Gag (p55) was detected in extracts programmed with 50 ng HIV-1 mRNA, but optimal translation was achieved with 100 ng (Figure 6D). Synthesis of Gag-Pol was not detected, probably because this polyprotein is synthesized by an inefficient mechanism involving ribosomal frameshifting [54] (data not shown). Pre-treatment with 20 ng of HIV-1 PR did not affect Gag production irrespective of the dose of HIV-1g mRNA used (Figure 6D). These results indicate that HIV-1g mRNA can be translated when eIF4GI and PABP are cleaved by HIV-1 PR.

To further reinforce this observation HeLa extracts were treated with increasing amounts of HIV-1 PR. After 30 min of pre-incubation, 50 or 100 ng HIV-1g mRNA were added to the translation mixture and protein synthesis was analyzed by autoradiography 1h later (Figure 6E). Translation of (+/+) luc mRNA was partially inhibited after incubation with 5 ng HIV-1 PR, and was almost completely blocked with 20 and 50 ng of the retroviral protease (Figure 6E and F). Notably, Gag polyprotein was synthesized from HIV-1g after pre-incubation with 5 or 20 ng HIV-1 PR in a similar amount than control samples, although an inhibition was observed (about 50%) when 50 ng of this protease was used (Figure 6E and F). The behaviour of HIV-1g mRNA in presence of HIV-1 PR was the same irrespective of the dose of mRNA used (Figure 6E). These results suggest that HIV-1g mRNA translation is more resistant to HIV-1 activity than cellular capped and polyadenylated mRNAs.

## Discussion

Host mRNAs are capped and polyadenylated by the cellular machinery. These structures are essential for mRNAs to be recognized by the protein synthesis machinery. Cleavage of translation initiation factors is a mechanism employed by a number of animal viruses to modulate host and viral protein synthesis [3]. In this regard, different viruses such as retroviruses, picornaviruses and caliciviruses have evolved similar strategies to interfere with cap and poly(A)-tail recognition by initiation factors, thereby maximizing the competitiveness of their own mRNAs for the translational machinery [2], [3]. eIF4GI, eIF4GII and PABP are targets for viral proteases in mammalian cells infected with some virus species, impairing the canonical initiation of translation [3], [9], [10], [17], [18], [55], [56], [57], [58]. Our present findings indicate that HIV-1 PR strongly inhibits translation of cellular mRNAs engaged with protein synthesis machinery. Furthermore, this protease blocks translation of polyadenylated mRNAs to a greater extent than their unpolyadenylated counterparts. These results have been observed in cap-poly(A) synergistic extracts (HeLa and Krebs-2 lysates), revealing that these proteases are able to abrogate synergism between the cap and poly(A) tail. In particular, translation of a capped and polyadenylated luc mRNA in HIV-1 PR-treated extracts decreased to the translational level of an uncapped and unpolyadenylated luc mRNA, suggesting that the cap and poly(A) tail do not contribute to translation when the protein synthesis machinery is modified by this viral protease.

Cleavage of both eIF4GI and eIF4GII is required for PV 2A^pro^ to inhibit completely the initiation of translation in HeLa cells [43]. Indeed, eIF4GI proteolysis alone is insufficient to block endogenous protein synthesis [43], [59]. These previous reports point to the idea that, apart from eIF4GI, HIV-1 PR may cleave an additional translation factor to abolish protein synthesis. We recently described that HIV-1 PR efficiently bisects PABP [16], as occurs with PV 3C^pro^ [18]. Both proteases separate NTD and CTD domains of PABP, but the retroviral protease carries out an additional cleavage within RRM3 [16]. As occurs in the case of 3C^pro^ [19], HIV-1 PR efficiently inhibits ongoing cellular translation, perhaps due to eIF4GI and PABP cleavage since both factor are essential to circularize mRNAs [30]. In addition, HIV-1 PR specifically blocks poly(A)-dependent initiation of translation. Thus, luciferase synthesis from an uncapped and polyadenylated mRNA is impaired in HIV-1 PR treated HeLa and Krebs-2 extracts, whereas a weak effect was observed on translation of an uncapped and unpolyadenylated luc mRNA. Moreover, HIV-1 PR has no apparent effect on Luc synthesis from both types of uncapped mRNAs in RRL, that does not exhibit poly(A)-dependent stimulation of translation. In addition, incubation with exogenous GST-PABP1 partially restores poly(A)-dependent translation in HIV-1 PR treated HeLa extracts. Probably, translation of polyadenylated mRNAs is not completely restored under our experimental conditions because eIF4GI is partially proteolyzed, and this proteolysis impedes the interaction between eIF4GI and PABP. Thus, simultaneous cleavage of eIF4GI and PABP might cooperate to block efficiently poly(A)-dependent translation underlying a double-target mechanism to inhibit host translation.

Kahvejian *et al.* reported that a recombinant PABP containing only the NTD portion is sufficient to significantly restore poly(A)-dependent translation in PABP depleted Krebs-2 extracts, since NTD contains the domains required to circularize the mRNA: RRMs and eIF4G interacting site [30]. Nevertheless, under their experimental conditions CTD was not present. CTD, separated by HIV-1 PR, could reduce the availability of translation factors such as eIF4B, Paip-1 or eRF3. Alternatively, lack of CTD could inhibit the oligomerization of PABP on poly(A) tail [3], [20]. HIV-1 PR also cleaves PABP within RRM3, rendering a product containing RRM1-2 [16]. A recombinant PABP protein that only contains RRM1-2 has little capacity to restore poly(A)-dependent translation in PABP-depleted Krebs-2 extracts [30]. In addition, cleavage products derived from PABP proteolysis by HIV-1 PR seem to be unstable in cultured cells or in cell extracts [16]. Therefore, HIV-1 PR strongly inactivates PABP for translation. In conclusion, we suggest that proteolysis of eIF4GI and PABP by HIV-1 PR blocks host protein synthesis, as occurs when eIF4GI and eIF4GII are cleaved by PV-2A^pro^. In this regard, PV-3C^pro^ provoked a potent inhibition of ongoing protein synthesis by PABP hydrolysis. This translational blockade increases when PV 3C^pro^ is combined with PV 2A^pro^, leading to both PABP and eIF4G inactivation [19]. Therefore, HIV-1 PR possesses some 2A^pro^ and 3C^pro^ activities, targeting both eIF4G and PABP [9], [16].

According to a recent report, PABP cleavage by 3C^pro^ inhibits PV IRES-driven translation [60]. It has been proposed that PABP cleavage, together with hydrolysis of other RNA-binding proteins, such as polypyrimidine tract-binding protein (PTB) and other PABP-associated proteins, are implicated in viral RNA switching from translation to replication [58], [60], [61], [62]. Translation of picornavirus IRES-containing mRNAs are stimulated by poly(A) tail, as occurs with host mRNAs. However, hydrolysis of both eIF4G and PABP takes place in PV-infected cells, suggesting that cap- and poly(A)-dependent translation should be impaired for both viral and host mRNAs. Cleavage of eIF4GI by HIV-1 PR did not enhance EMCV and PV IRES-driven translation to a level comparable to that found after 2A^pro^ incubation, although it was sufficient to replace IRES poly(A) tail synergism in HeLa extracts. In agreement with this data, 2A^pro^ partially restores PV IRES-driven translation in HeLa extracts treated with 3C^pro^ [60]. Therefore, HIV-1 PR resembles to some extent the action of 2A^pro^ and 3C^pro^ on picornavirus IRES-driven translation, perhaps due to simultaneous cleavage of eIF4GI and PABP.

Early findings indicated that HIV-1 mRNAs are translated by a cap-dependent mechanism as occurs with most cellular mRNAs [63]. However, recent reports have proposed that HIV-1 mRNAs as well as mRNAs from other lentiviruses could be translated by internal initiation [12], [13], [14], [15], [47], [64]. In addition, a dual mechanism (cap-dependent and cap-independent) translation has been proposed for HIV-1 mRNAs [65]. Leader sequences from HIV-1 mRNAs are long and highly structured, rendering HIV-1 mRNAs a poor substrate for ribosome scanning and cap-dependent translation. In addition, the leader sequence of HIV-1 and HIV-2 contains encapsidation signals, which are recognized by Gag polyprotein. Oligomerization of Gag polyprotein onto HIV-1 and HIV-2 leader sequence may inhibit the scanning of the initiation complex [51], [64]. Thus, internal initiation could be a plausible mechanism for direct recruitment of ribosomes to the initiation AUG during HIV infection. Consistent with this idea, our present findings reveal that Gag polyprotein is synthesized in spite of eIF4GI and PABP cleavage by HIV-1 PR from an authentic HIV-1g mRNA. These results indicate that HIV-1g mRNA can be translated when cap- and poly(A)-dependent translation is arrested, supporting the concept that translation initiation of this mRNA proceeds by a non-canonical mechanism. We previously described that the translation of an unpolyadenylated luc reporter mRNA bearing the HIV-1 leader sequence (cap-L5′HIV-Luc mRNA) is inhibited by HIV-1 PR in HeLa extracts, whereas Gag synthesis from an mRNA that also contains *gag* gene (cap-L5′GAG-PR mRNA) is stimulated under these conditions [9], [47]. In this regard, we analyze here the translatability of an authentic polyadenylated HIV-1 genomic mRNA in presence of the retroviral protease. The data presented here using HIV-1g mRNA agree well with the experiment carried out with cap-L5′GAG-PR mRNA in HeLa extracts [9], [47], supporting the idea that both mRNAs are efficiently translated in presence of HIV-1 PR. Further efforts are necessary to determine the exact molecular mechanism by which HIV-1 mRNAs are engaged in the protein synthesis machinery in HIV-1 infected cells.

## Supporting Information

Figure S1 Translation of reporter luc mRNAs in HeLa extracts. A) HeLa extracts were programmed with 50 ng (+/−), (+/+), (−/−) and (−/+) luc mRNAs. 1 h later luciferase activity was measured in each case and relative luciferase activity from three independent experiments was plotted. B) HeLa extracts were programmed with 50 ng (+/G/−), (+/G/+), (−/G/−) and (−/G/+) luc mRNAs. 1 h later luciferase activity was measured in each case and relative luciferase activity from three independent experiments was plotted. C) HeLa extracts were programmed with 50 ng (+/+) mRNA and luciferase activity was analyzed after 5, 10 and 15 min. Error bars represent SD from two independent experiments.
(0.20 MB JPG)

Figure S2 Translation of exogenous and endogenous luc mRNAs in HIV-1 PR treated Kreb-2 extracts. Non-nuclease-treated Kreb-2 extracts supplemented with [35S]Met-[35S]Cys/ml were programmed with 50 ng of (+/−) or (+/+) luc mRNAs. After 8 min, 20 ng of HIV-1 PR were added to the lysates. The samples were analyzed 1 and 3 h after the initiation of the reaction. A) Luc activity at each time point was measured and plotted. Error bars indicate standard deviations obtained from three measurements of each sample. B) Relative quantification of the Luc activity obtained from HeLa extracts programmed with (+/−) or (+/+) mRNAs in presence of HIV-1 PR with respect to control extracts after 1 h of incubation. SDs were obtained from three independent experiments. C) eIF4GI, eIF4GII and PABP were detected by western blot. D) Endogenous protein synthesis was analyzed by SDS-PAGE followed by fluorography and autoradiography.
(0.24 MB JPG)

## References

[1] Schneider RJ, Mohr I (2003). Translation initiation and viral tricks.. Trends Biochem Sci.

[2] Prevot D, Darlix JL, Ohlmann T (2003). Conducting the initiation of protein synthesis: the role of eIF4G.. Biol Cell.

[3] Lloyd RE (2006). Translational control by viral proteinases.. Virus Res.

[4] Gingras AC, Raught B, Sonenberg N (1999). eIF4 initiation factors: effectors of mRNA recruitment to ribosomes and regulators of translation.. Annu Rev Biochem.

[5] Imataka H, Gradi A, Sonenberg N (1998). A newly identified N-terminal amino acid sequence of human eIF4G binds poly(A)-binding protein and functions in poly(A)-dependent translation.. Embo J.

[6] Mader S, Lee H, Pause A, Sonenberg N (1995). The translation initiation factor eIF-4E binds to a common motif shared by the translation factor eIF-4 gamma and the translational repressors 4E-binding proteins.. Mol Cell Biol.

[7] Gallie DR (1998). A tale of two termini: a functional interaction between the termini of an mRNA is a prerequisite for efficient translation initiation.. Gene.

[8] Alvarez E, Menendez-Arias L, Carrasco L (2003). The eukaryotic translation initiation factor 4GI is cleaved by different retroviral proteases.. J Virol.

[9] Ventoso I, Blanco R, Perales C, Carrasco L (2001). HIV-1 protease cleaves eukaryotic initiation factor 4G and inhibits cap-dependent translation.. Proc Natl Acad Sci U S A.

[10] Gradi A, Svitkin YV, Imataka H, Sonenberg N (1998). Proteolysis of human eukaryotic translation initiation factor eIF4GII, but not eIF4GI, coincides with the shutoff of host protein synthesis after poliovirus infection.. Proc Natl Acad Sci U S A.

[11] Martinez-Salas E, Fernandez-Miragall O (2004). Picornavirus IRES: structure function relationship.. Curr Pharm Des.

[12] Brasey A, Lopez-Lastra M, Ohlmann T, Beerens N, Berkhout B (2003). The leader of human immunodeficiency virus type 1 genomic RNA harbors an internal ribosome entry segment that is active during the G2/M phase of the cell cycle.. J Virol.

[13] Buck CB, Shen X, Egan MA, Pierson TC, Walker CM (2001). The human immunodeficiency virus type 1 gag gene encodes an internal ribosome entry site.. J Virol.

[14] Camerini V, Decimo D, Balvay L, Pistello M, Bendinelli M (2008). A dormant internal ribosome entry site controls translation of feline immunodeficiency virus.. J Virol.

[15] Herbreteau CH, Weill L, Decimo D, Prevot D, Darlix JL (2005). HIV-2 genomic RNA contains a novel type of IRES located downstream of its initiation codon.. Nat Struct Mol Biol.

[16] Alvarez E, Castello A, Menendez-Arias L, Carrasco L (2006). HIV protease cleaves poly(A)-binding protein.. Biochem J.

[17] Kuyumcu-Martinez M, Belliot G, Sosnovtsev SV, Chang KO, Green KY (2004). Calicivirus 3C-like proteinase inhibits cellular translation by cleavage of poly(A)-binding protein.. J Virol.

[18] Kuyumcu-Martinez NM, Joachims M, Lloyd RE (2002). Efficient cleavage of ribosome-associated poly(A)-binding protein by enterovirus 3C protease.. J Virol.

[19] Kuyumcu-Martinez NM, Van Eden ME, Younan P, Lloyd RE (2004). Cleavage of poly(A)-binding protein by poliovirus 3C protease inhibits host cell translation: a novel mechanism for host translation shutoff.. Mol Cell Biol.

[20] Rivera CI, Lloyd RE (2008). Modulation of enteroviral proteinase cleavage of poly(A)-binding protein (PABP) by conformation and PABP-associated factors.. Virology.

[21] Rodriguez Pulido M, Serrano P, Saiz M, Martinez-Salas E (2007). Foot-and-mouth disease virus infection induces proteolytic cleavage of PTB, eIF3a,b, and PABP RNA-binding proteins.. Virology.

[22] Cheng S, Gallie DR (2007). eIF4G, eIFiso4G, and eIF4B bind the poly(A)-binding protein through overlapping sites within the RNA recognition motif domains.. J Biol Chem.

[23] Cosson B, Berkova N, Couturier A, Chabelskaya S, Philippe M (2002). Poly(A)-binding protein and eRF3 are associated in vivo in human and Xenopus cells.. Biol Cell.

[24] Le H, Tanguay RL, Balasta ML, Wei CC, Browning KS (1997). Translation initiation factors eIF-iso4G and eIF-4B interact with the poly(A)-binding protein and increase its RNA binding activity.. J Biol Chem.

[25] Uchida N, Hoshino S, Imataka H, Sonenberg N, Katada T (2002). A novel role of the mammalian GSPT/eRF3 associating with poly(A)-binding protein in Cap/Poly(A)-dependent translation.. J Biol Chem.

[26] Craig AW, Haghighat A, Yu AT, Sonenberg N (1998). Interaction of polyadenylate-binding protein with the eIF4G homologue PAIP enhances translation.. Nature.

[27] Khaleghpour K, Kahvejian A, De Crescenzo G, Roy G, Svitkin YV (2001). Dual interactions of the translational repressor Paip2 with poly(A) binding protein.. Mol Cell Biol.

[28] Khaleghpour K, Svitkin YV, Craig AW, DeMaria CT, Deo RC (2001). Translational repression by a novel partner of human poly(A) binding protein, Paip2.. Mol Cell.

[29] Kahvejian A, Roy G, Sonenberg N (2001). The mRNA closed-loop model: the function of PABP and PABP-interacting proteins in mRNA translation.. Cold Spring Harb Symp Quant Biol.

[30] Kahvejian A, Svitkin YV, Sukarieh R, M'Boutchou MN, Sonenberg N (2005). Mammalian poly(A)-binding protein is a eukaryotic translation initiation factor, which acts via multiple mechanisms.. Genes Dev.

[31] Aldabe R, Feduchi E, Novoa I, Carrasco L (1995). Expression of poliovirus 2Apro in mammalian cells: effects on translation.. FEBS Lett.

[32] Barco A, Feduchi E, Carrasco L (2000). A stable HeLa cell line that inducibly expresses poliovirus 2A(pro): effects on cellular and viral gene expression.. J Virol.

[33] Ventoso I, Barco A, Carrasco L (1998). Mutational analysis of poliovirus 2Apro. Distinct inhibitory functions of 2apro on translation and transcription.. J Biol Chem.

[34] Ventoso I, Carrasco L (1995). A poliovirus 2A(pro) mutant unable to cleave 3CD shows inefficient viral protein synthesis and transactivation defects.. J Virol.

[35] Burgui I, Aragon T, Ortin J, Nieto A (2003). PABP1 and eIF4GI associate with influenza virus NS1 protein in viral mRNA translation initiation complexes.. J Gen Virol.

[36] Cuconati A, Molla A, Wimmer E (1998). Brefeldin A inhibits cell-free, de novo synthesis of poliovirus.. J Virol.

[37] Franco D, Pathak HB, Cameron CE, Rombaut B, Wimmer E (2005). Stimulation of poliovirus synthesis in a HeLa cell-free in vitro translation-RNA replication system by viral protein 3CDpro.. J Virol.

[38] Molla A, Paul AV, Wimmer E (1991). Cell-free, de novo synthesis of poliovirus.. Science.

[39] Aviv H, Boime I, Leder P (1971). Protein synthesis directed by encephalomyocarditis virus RNA: properties of a transfer RNA-dependent system.. Proc Natl Acad Sci U S A.

[40] Svitkin YV, Ginevskaya VA, Ugarova TY, Agol VI (1978). A cell-free model of the encephalomyocarditis virus-induced inhibition of host cell protein synthesis.. Virology.

[41] Svitkin YV, Sonenberg N (2004). An efficient system for cap- and poly(A)-dependent translation in vitro.. Methods Mol Biol.

[42] Castello A, Sanz MA, Molina S, Carrasco L (2006). Translation of Sindbis virus 26S mRNA does not require intact eukariotic initiation factor 4G.. J Mol Biol.

[43] Castello A, Alvarez E, Carrasco L (2006). Differential cleavage of eIF4GI and eIF4GII in mammalian cells. Effects on translation.. J Biol Chem.

[44] Aldabe R, Feduchi E, Novoa I, Carrasco L (1995). Efficient cleavage of p220 by poliovirus 2Apro expression in mammalian cells: effects on vaccinia virus.. Biochem Biophys Res Commun.

[45] Novoa I, Martinez-Abarca F, Fortes P, Ortin J, Carrasco L (1997). Cleavage of p220 by purified poliovirus 2A(pro) in cell-free systems: effects on translation of capped and uncapped mRNAs.. Biochemistry.

[46] Novoa I, Carrasco L (1999). Cleavage of eukaryotic translation initiation factor 4G by exogenously added hybrid proteins containing poliovirus 2Apro in HeLa cells: effects on gene expression.. Mol Cell Biol.

[47] Perales C, Carrasco L, Ventoso I (2003). Cleavage of eIF4G by HIV-1 protease: effects on translation.. FEBS Lett.

[48] Michel YM, Poncet D, Piron M, Kean KM, Borman AM (2000). Cap-Poly(A) synergy in mammalian cell-free extracts. Investigation of the requirements for poly(A)-mediated stimulation of translation initiation.. J Biol Chem.

[49] Michel YM, Borman AM, Paulous S, Kean KM (2001). Eukaryotic initiation factor 4G-poly(A) binding protein interaction is required for poly(A) tail-mediated stimulation of picornavirus internal ribosome entry segment-driven translation but not for X-mediated stimulation of hepatitis C virus translation.. Mol Cell Biol.

[50] Svitkin YV, Imataka H, Khaleghpour K, Kahvejian A, Liebig HD (2001). Poly(A)-binding protein interaction with elF4G stimulates picornavirus IRES-dependent translation.. Rna.

[51] Anderson EC, Lever AM (2006). Human immunodeficiency virus type 1 Gag polyprotein modulates its own translation.. J Virol.

[52] Groom HC, Anderson EC, Dangerfield J, Lever AM (2009). Rev regulates translation of Human Immunodeficiency Virus Type-1 RNAs.. J Gen Virol.

[53] Perales C, Carrasco L, Gonzalez ME (2005). Regulation of HIV-1 env mRNA translation by Rev protein.. Biochim Biophys Acta.

[54] Wilson W, Braddock M, Adams SE, Rathjen PD, Kingsman SM (1988). HIV expression strategies: ribosomal frameshifting is directed by a short sequence in both mammalian and yeast systems.. Cell.

[55] Gradi A, Foeger N, Strong R, Svitkin YV, Sonenberg N (2004). Cleavage of eukaryotic translation initiation factor 4GII within foot-and-mouth disease virus-infected cells: identification of the L-protease cleavage site in vitro.. J Virol.

[56] Joachims M, Van Breugel PC, Lloyd RE (1999). Cleavage of poly(A)-binding protein by enterovirus proteases concurrent with inhibition of translation in vitro.. J Virol.

[57] Willcocks MM, Carter MJ, Roberts LO (2004). Cleavage of eukaryotic initiation factor eIF4G and inhibition of host-cell protein synthesis during feline calicivirus infection.. J Gen Virol.

[58] Zhang B, Morace G, Gauss-Muller V, Kusov Y (2007). Poly(A) binding protein, C-terminally truncated by the hepatitis A virus proteinase 3C, inhibits viral translation.. Nucleic Acids Res.

[59] Gradi A, Imataka H, Svitkin YV, Rom E, Raught B (1998). A novel functional human eukaryotic translation initiation factor 4G.. Mol Cell Biol.

[60] Bonderoff JM, Larey JL, Lloyd RE (2008). Cleavage of poly(A)-binding protein by poliovirus 3C proteinase inhibits viral internal ribosome entry site-mediated translation.. J Virol.

[61] Back SH, Kim YK, Kim WJ, Cho S, Oh HR (2002). Translation of polioviral mRNA is inhibited by cleavage of polypyrimidine tract-binding proteins executed by polioviral 3C(pro).. J Virol.

[62] de Breyne S, Bonderoff JM, Chumakov KM, Lloyd RE, Hellen CU (2008). Cleavage of eukaryotic initiation factor eIF5B by enterovirus 3C proteases.. Virology.

[63] Miele G, Mouland A, Harrison GP, Cohen E, Lever AM (1996). The human immunodeficiency virus type 1 5′ packaging signal structure affects translation but does not function as an internal ribosome entry site structure.. J Virol.

[64] Ricci EP, Herbreteau CH, Decimo D, Schaupp A, Datta SA (2008). In vitro expression of the HIV-2 genomic RNA is controlled by three distinct internal ribosome entry segments that are regulated by the HIV protease and the Gag polyprotein.. Rna.

[65] Ricci EP, Soto Rifo R, Herbreteau CH, Decimo D, Ohlmann T (2008). Lentiviral RNAs can use different mechanisms for translation initiation.. Biochem Soc Trans.

