# Supplementary figures and images for: HIV- 1 Protease Inhibits Cap- and Poly(A)-Dependent Translation upon eIF4GI and PABP Cleavage
Source: PLoS One. 2009 Nov 24;4(11):e7997. doi: 10.1371/journal.pone.0007997 (PMC2776998; doi:10.1371/journal.pone.0007997)

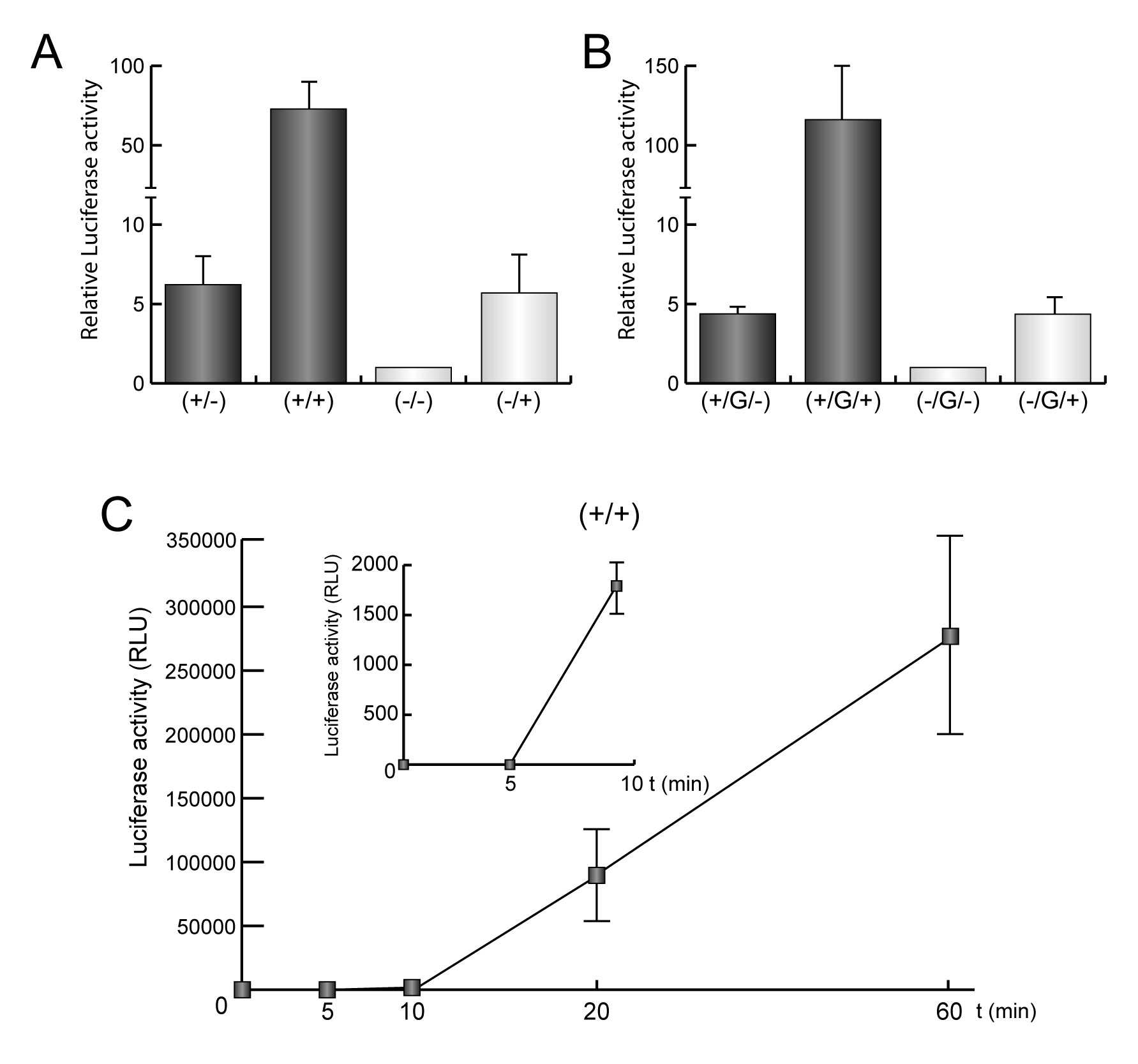

Supplement: Figure S1 — Translation of reporter luc mRNAs in HeLa extracts. A) HeLa extracts were programmed with 50 ng (+/−), (+/+), (−/−) and (−/+) luc mRNAs. 1 h later luciferase activity was measured in each case and relative luciferase activity from three independent experiments was plotted. B) HeLa extracts were programmed with 50 ng (+/G/−), (+/G/+), (−/G/−) and (−/G/+) luc mRNAs. 1 h later luciferase activity was measured in each case and relative luciferase activity from three independent experiments was plotted. C) HeLa extracts were programmed with 50 ng (+/+) mRNA and luciferase activity was analyzed after 5, 10 and 15 min. Error bars represent SD from two independent experiments. (0.20 MB JPG) [file pone.0007997.s001.jpg]

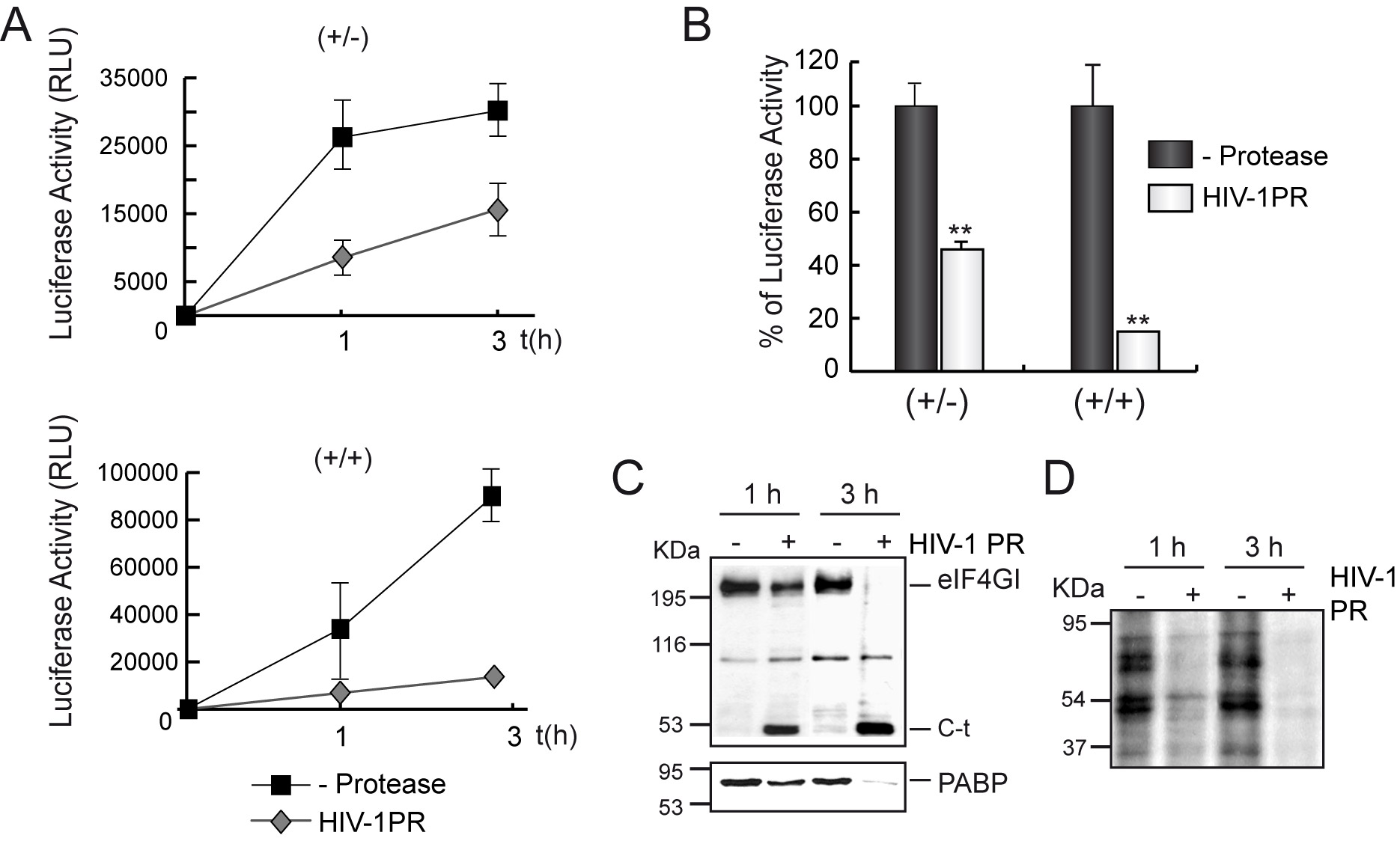

Supplement: Figure S2 — Translation of exogenous and endogenous luc mRNAs in HIV-1 PR treated Kreb-2 extracts. Non-nuclease-treated Kreb-2 extracts supplemented with [35S]Met-[35S]Cys/ml were programmed with 50 ng of (+/−) or (+/+) luc mRNAs. After 8 min, 20 ng of HIV-1 PR were added to the lysates. The samples were analyzed 1 and 3 h after the initiation of the reaction. A) Luc activity at each time point was measured and plotted. Error bars indicate standard deviations obtained from three measurements of each sample. B) Relative quantification of the Luc activity obtained from HeLa extracts programmed with (+/−) or (+/+) mRNAs in presence of HIV-1 PR with respect to control extracts after 1 h of incubation. SDs were obtained from three independent experiments. C) eIF4GI, eIF4GII and PABP were detected by western blot. D) Endogenous protein synthesis was analyzed by SDS-PAGE followed by fluorography and autoradiography. (0.24 MB JPG) [file pone.0007997.s002.jpg]
